# Lessons from COVID-19 pandemic for the child survival agenda

**DOI:** 10.7189/jogh.10.020357

**Published:** 2020-12

**Authors:** S V Subramanian, Pritha Chatterjee, Omar Karlsson

**Affiliations:** 1 Harvard Center for Population and Development Studies, Cambridge, Massachusetts, USA; 2 Department of Social and Behavioral Sciences, Harvard T.H. Chan School of Public Health, Boston, Massachusetts, USA; 3 Takemi Program in International Health, Harvard T.H. Chan School of Public Health, Boston, Massachusetts, USA

The public discourse around the COVID-19 pandemic has been strikingly quantitative. Worldwide, the mainstream media has regularly informed the public of confirmed COVID-19 cases and deaths, including projections of worst-case scenarios drawn from esoteric epidemiological models. The prominence and visibility of data, regardless of its completeness or quality, underscored the threat of COVID-19 to policy makers and lay individuals alike. It also prompted governments to swiftly lock down their societies, despite the socioeconomic disruptions and human suffering associated with such lockdowns. The widespread media coverage of COVID-19 data and swift response from governments highlight the potency of *real-time data*, and contain important lessons for public health policy, which when applied, could raise the profile of other health issues and spur action among key stakeholders.

## LESSONS FOR THE GLOBAL CHILD SURVIVAL AGENDA

The dire absence of similar real-time data on other important population health indicators is striking. Consider deaths among *children under the age of five*; the commonly used metric for monitoring progress in child survival. Globally, more than 5 million children died in 2018; unfortunately, the most recent year for which child death data are publicly available. Furthermore, that the latest child mortality rate per 1000 live births ranges from 5 in high income countries to 49 in lower middle income countries and 68 in low income countries, suggests that the vast majority of these deaths were preventable [1,2].

**Figure 1:**
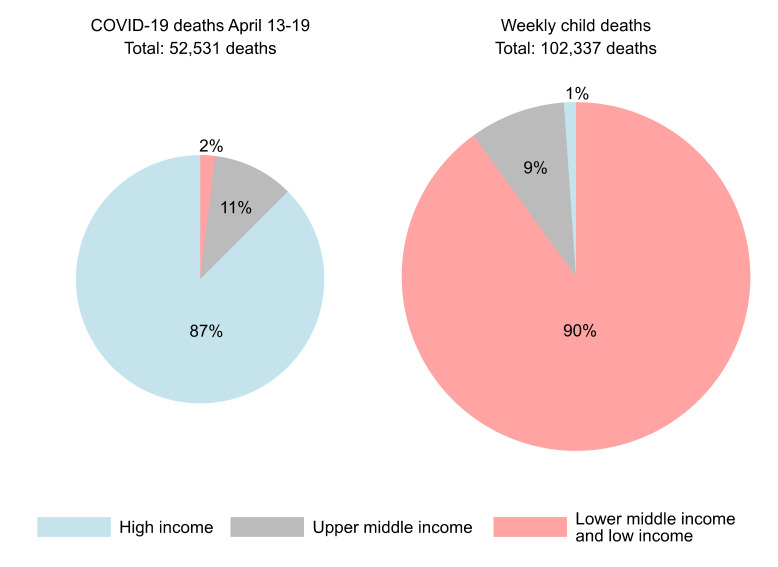
: Distribution of global child deaths/per week (2018) and COVID-19 deaths for the worst week (April 13-19, 2020) across World Bank income classification. Data sources: [1-3].

To put these statistics in the current context of COVID-19 fatalities, 103 684 children (5 391 563 deaths/52 weeks) died every week in 2018. This figure is significantly greater than the COVID-19 deaths during the deadliest week yet of the pandemic, when 52 531 people died (**Figure 1**) [3]. The disparity in overall deaths holds when viewed at a national level too. In India, for instance, 16 961 children died every week in 2018, which is more than double that or India’s worst week yet of COVID-19 fatalities (n = 6925, August 24-30, 2020) [2,3]. COVID-19 deaths may well be underreported, but the same is true for child deaths, especially in low income countries.

If public action were dictated by the sheer magnitude of deaths, then the child survival agenda should have overwhelmingly pre-occupied policy thinking and action. Over 60 million children are estimated to have died just in the past decade, and some 46 million more are projected to die, in the coming decade [1]. While substantial work continues globally to address childhood deaths, the issue has not evoked the same sense of urgency, purpose and response that the world witnessed with regards to COVID-19, especially in terms of media coverage. This is despite the experience and knowledge from century-old successful public health interventions to reduce preventable child mortality [4].

**Figure:**
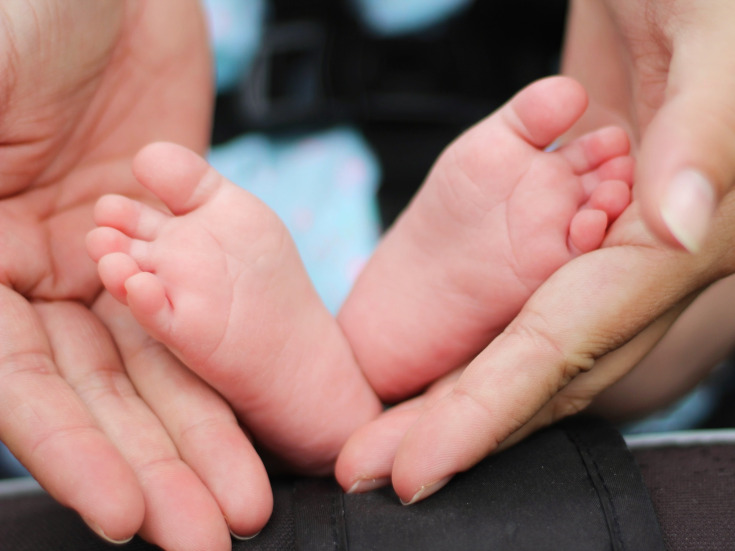
Photo: Photo by Bonnie Kittle on Unsplash, via https://unsplash.com/photos/-f7bKsvOgwU.

## REAL-TIME DATA IN PUBLIC DOMAIN IS EMPOWERING

The COVID-19 crisis has shown the power of publicly available real-time data to drive the news cycle, inform policy makers, and raise public awareness of public health priorities. Many low- and middle-income countries, such as India, have managed to collect and release data on COVID-19 statistics with consistent frequency. Yet, the latest available data on the number of child deaths in India is at least 2 years old [1,2].

In most low- and middle-income countries there is no *true* count of child deaths available, in most LMICs, because not all child deaths are actually counted. What are reported are “statistical estimates” arrived through surveying a sample of mothers and their recollection of the death history of all the children they have ever had. Robust civil registration systems are crucial for bringing accountability and equity to public health and polity. Sample-based estimates are, indeed, useful. However, they have failed to galvanize policy makers, politicians and the public to action.

In an incredibly short period of time, organizations have swiftly developed systems and networks for reporting COVID-19 statistics. The pandemic has shown us that when the public demand for data are real and urgent, non-governmental institutions voluntarily step in to fill the void. It would indeed be a crisis wasted if we fail to capitalize on these data synergies and to adept them for reporting child deaths on a daily, weekly, or even monthly basis. The COVID-19 experience suggests that this should stimulate mainstream media to constructively inform and push political leadership and governments to show similar urgency and purpose to the agenda of child survival.

While the availability of real-time updates around COVID-19 has helped journalists underscore its public health importance on a daily basis, for most public health issues, when news fatigue sets in, or when other, competing newsworthy issues come up, the media’s attention is likely to be diverted. This is particularly true of “routine” public health issues, which may not be as rare or as urgent as a global pandemic. At the same time, the role of media in this pandemic – catalyzed through public availability of real time data – has been instrumental in also contributing to the discussion on establishing robust epidemiologic data surveillance and transparent sharing of data.

Initial data science efforts in this direction will undoubtedly have its limitations; but, as we have learned from the reporting of public health and statistics departments around the world during this pandemic, data begets better data. A real-time data science initiative to count child deaths will further catalyze efforts to enumerating every child death. Such timely tracking could also help us better understand how policies to mitigate child mortality are performing in real time and increase accountability among policy makers, legislators and practicioners. In time, other critical population and health indicators could be added.

Itt is critical to remember the striking global asymmetry in child deaths. While 1% of the global child deaths are in high income countries [3], low- and lower middle-income countries account for 90% of all child deaths (**Figure 1**) [3]. In the early days of the pandemic in April, an asymmetry was also seen in the COVID-19 death toll, albeit in the reverse direction, with high income countries accounting for the vast majority of the global COVID-19 deaths. However, as the pandemic is evolving, this asymmetry has become less stark, though it is still present.

If COVID-19 had not affected the high-income countries in the manner it did, would the response from scientists, media and policy makers have had the same urgency and sense of purpose? Indeed, as COVID-19 deaths move away from high-income countries, we will see whether the intense focus on COVID-19 will remain. A recent analysis of news coverage found that 41 000 English print news stories and 19 000 headlines included the word “Coronavirus” in the first month of the COVID-19 pandemic. In comparison, during the first month of the 2018 Ebola outbreak in the Democratic Republic of Congo, there were only 1800 English articles and 700 headlines mentioning the crisis [5].

Leading dailies have recently paid tributes to COVID-19 victims in the United States drawing attention to the real lives behind the statistics, with headlines such as ‘*An Incalculable Loss’* and ‘*Faces of the Dead*’. It is unfortunate that we have to use a global pandemic with colossal loss of lives to draw attention to another recurring silent tragedy – childhood deaths. Creating a publicly available real-time data infrastructure to track child deaths is well worth the effort if it will spur action that accelerates the global elimination of preventable child deaths. Mr. Bill Gates, who has championed the cause of reducing global child mortality through his philanthropic work, recently voiced his concern on prematurely lifting of a lockdown that was put in place to counter COVID-19, stating that while the economy might re-open sooner no one would be able to “*ignore that pile of bodies over in the corner*” [6]. It will serve us well to remember that sentiment when 100 000 children are dying every week.
